# Experimental quantum state certification by actively sampling photonic entangled states

**DOI:** 10.1126/sciadv.aea4144

**Published:** 2026-02-11

**Authors:** Michael Antesberger, Mariana M. E. Schmid, Huan Cao, Borivoje Dakić, Lee A. Rozema, Philip Walther

**Affiliations:** ^1^ University of Vienna, Faculty of Physics, Vienna Center for Quantum Science and Technology (VCQ) and Research platform TURIS, Boltzmanngasse 5, 1090 Vienna, Austria.; ^2^ Christian Doppler Laboratory for Photonic Quantum Computer, Faculty of Physics, University of Vienna, 1090 Vienna, Austria.; ^3^ Institute for Quantum Optics and Quantum Information (IQOQI) Vienna, Austrian Academy of Sciences, Boltzmanngasse 3, Vienna, Austria.

## Abstract

Entangled quantum states are essential ingredients for many quantum technologies, but they must be validated before they are used. As a full characterization is prohibitively resource intensive, recent work has focused on developing methods to efficiently extract a few parameters of interest, in a so-called verification framework. Most existing approaches are based on preparing an ensemble of nominally identical and independently distributed (IID) quantum states and then measuring each copy of the ensemble. However, this leaves no states left for the intended quantum tasks and the IID assumptions do not always hold experimentally. To overcome these challenges, we experimentally implement quantum state certification (QSC), which measures only a subset of the ensemble, certifying the fidelity of multiple copies of the remaining states. We use active optical switches to randomly sample from sources of two-photon Bell states and three-photon GHZ (Greenberger-Horn-Zeilinger) states, reporting statistically sound fidelities in real time without destroying the entire ensemble. In addition, our QSC protocol removes the assumption that the states are identically distributed (but still assumes independent copies); can achieve close *N*^−1^ scaling, in the number of states measured *N*; and can be implemented in a device-independent manner. Together, these benefits make our QSC protocol suitable for benchmarking large-scale quantum computing devices and deployed quantum communication setups relying on entanglement in both standard and adversarial situations.

## Teaser

Quantum state certification is implemented with optimal scaling without consuming all the copies.

## INTRODUCTION

Quantum technology uses entangled states as resources to implement tasks with an efficiency or security that cannot be accomplished with only classical resources (*1*, *2*). However, before using an entangled state for a given task, the experimentally produced state must be verified. Traditionally, this is done by preparing an ensemble of *N* identical and independently distributed (IID) states and measuring each state. It is widely appreciated that learning the complete quantum state—i.e., performing quantum state tomography (*3*)—is a challenging task, requiring *N* to increase exponentially with the system size. This has led to a variety of resource efficient characterization methods, including adaptive state tomography (*4*–*6*), compressed sensing (*7*), direct fidelity estimation (*8*), cross-verification (*9*, *10*), and quantum state verification (QSV) (*11*–*20*). In all of these approaches, one measures every copy of the initially prepared ensemble, leaving no states for use in a further experiment. One must therefore assume that the source operates identically during the characterization and operation phases. Here, we circumvent this problem by experimentally implementing a quantum state certification (QSC) protocol proposed by Gočanin *et al.* (*21*).

In QSC, only a subset of the ensemble is measured, allowing one to certify some property of the remaining states. In more detail, we imagine a “verifier” who randomly extracts a subset of the initial ensemble of physical states and a “user” who receives the remaining states. The verifier performs QSV on their subensemble, allowing her to issue a certificate to the user that his remaining states are close to a promised target state. QSC thus relies on QSV. More precisely, QSC considers a set of *N* independent (but not necessarily identically distributed) physical states. The verifier then extracts a random subset of μ*N* states, measuring each one and subsequently issues a certificate regarding the unmeasured states of the user. QSC then answers the question, “With what confidence can we conclude that the remaining subset of (1−μ)N physical states have an average fidelity of at least 95% with the target state?”

Both QSV and QSC are useful but have different potential applications. For example, QSV could be used in initial source characterizations or where the goal is simply to determine whether a given state has been successfully distributed through a quantum network. However, QSV consumes all of the states. Therefore, QSC would have applications when it is important to ensure that the state has been distributed but there is another goal or protocol to be implemented. In particular, the remaining states can be used for these other protocols.

QSC is experimentally accessible, meaning that all measurements can be made locally and the postprocessing complexity is low. This is because it is based on QSV, which can estimate the fidelity with the optimal *N*^−1^ scaling (*22*, *23*). Although much work on state characterization operates in a trusted, device-dependent scenario, there is a growing need to perform device-independent (DI) state characterization with untrusted measurement devices (*24*–*26*), for applications such as blind quantum computing (*16*, *17*, *27*) and quantum cryptography (*28*, *29*). Our work is based on the proposal by Gočanin *et al.* (*21*), which introduces DI-QSV and DI-QSC, allowing us to experimentally realize efficient QSC. Although, in our experiment, we do not close the loopholes necessary to claim device independence, we provide a detailed discussion of how to extend our experiment to a DI setting.

Photonic Bell states and Greenberger-Horn-Zeilinger (GHZ) states are of extreme importance for a variety of applications. In particular, two-photon Bell states are an essential primitive for many quantum communication protocols (*29*–*31*), whereas three-photon GHZ states and probabilistic fusion gates can enable the efficient generation of large-scale cluster states (*32*) for measurement-based quantum computing (*33*–*35*). Certifying such resources is essential for these and other applications. To show the applicability of our QSC protocol, we therefore experimentally implement it for both two-photon Bell states and three-photon GHZ states (*36*, *37*).

**Fig. 1.**
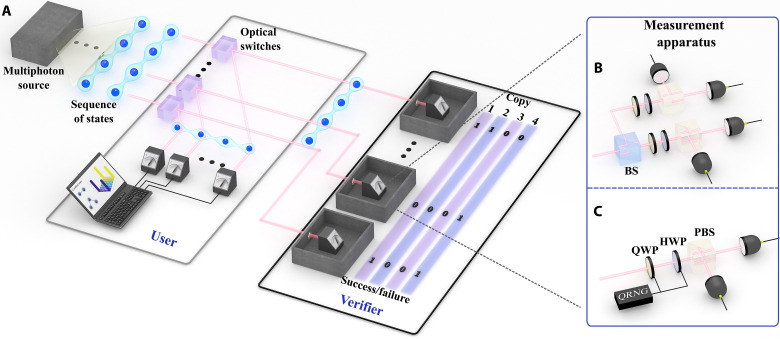
Experimental QSC. (**A**) Sequences of *M*-photon states are spontaneously produced and sent to *M* trusted synchronized OSs. The switches periodically alternate between two settings, directing the inputs to the user or verifier. We drive the switches faster than the photons are produced. Because the emission time is random, this serves to randomly send each state to the user or verifier. Both the user and verifier implement standard polarization measurements on their photons. (**B**) Two-photon QSV measurement. We randomly select the measurement basis for our two-photon experiment using 50:50 beam splitters to send each of the *M* photons to one of two polarization measurements. (**C**) Three-photon QSV measurement. For our three-photon experiment, because the three-photon rate is sufficiently low, we randomly select the measurement basis by rotating the waveplates between two settings. Each subsequent setting is chosen on the basis of the outcome of a commercial QRNG. Figure 1 was created using 3ds Max by H.C.

In our implementation of QSC, some of the produced quantum states are randomly routed to the verifier who performs QSV while the user simultaneously runs his experiment. The user can either adapt to the current confidence level broadcasted by the verifier or wait until sufficient confidence is achieved. If the verifier’s measurements are successful, the states arriving to user are certified. Note that our implementation is slightly different from the proposal in (*21*), wherein the user does not measure their copies until the verifier issues a report. However, because we do not have a quantum memory, our user and verifier operate simultaneously, making our implementation already applicable with today’s technology. To realize this deterministically, we use active optical switches (OSs) (*38*), as outlined in Fig. 1, because we use active switches; each individual state is deterministically routed to the user or verifier. This provides a realistic and practical implementation of QSC because, from the user’s point of view, the only effect is a constant reduction in the counting rate, which does not scale with the system size.

## RESULTS

### Device-independent quantum state certification

Our implementation of QSC is based on self-testing (*39*). Although the specifics depend on the target state, the idea of self-testing is that certain quantum correlations are almost unique to some target states. For example, self-testing for a GHZ state works as follows. One attempts to violate a Mermin inequality (*40*) with the physical states. This can only be achieved if the physical states are close to the target state. Then, if the violation is sufficiently strong, one can place bounds on the average fidelity $\bar{F}$ of the physical states. We mention that, in a DI framework, it is not possible to directly assess the fidelity. Instead, one uses the extractability Ξ, which is equivalent to the fidelity up to local isometries (*39*, *41*, *42*). However, for simplicity, in the following, we use fidelity *F* (or infidelity η=1−F) in place of the extractability if not otherwise specified. Because self-testing is based on the mean value of observables, it usually does not discuss the behavior for finite *N* (*39*, *43*). To work in this regime, QSV converts self-testing to a nonlocal game that can only be won by the target state (*21*).

To explain QSV, consider a source producing an independent sequence of *N* states $S=\left\{\sigma_{1},\sigma_{2},\ldots ,\sigma_{N}\right\}$ and a target state ∣ψ〉 that, for now, we take to be a three-photon GHZ state. The Mermin inequality for this target state is (*40*)

$$\begin{array}{c} B=\sum_{o_{1},o_{2},o_{3}}{\left(-1\right)}^{o_{1}+o_{2}+o_{3}}\left[p\left(o_{1},o_{2},o_{3}|0,0,1\right)+p(o_{1},o_{2},o_{3}|0,1,0)\right.\\ \left.+p(o_{1},o_{2},o_{3}|1,0,0)-p(o_{1},o_{2},o_{3}|1,1,1)\right]\leq 2 \end{array}$$ (1)

Here, the individual terms are probabilities of outcomes to be estimated experimentally. In more detail, $p\left(o_{1},o_{2},o_{3}|i_{1},i_{2},i_{3}\right)$ are the probabilities to obtain outcomes $o_{1}$, $o_{2}$, and $o_{3}$ when qubits one, two, and three are measured with a setting defined by $i_{1}$, $i_{2}$, and $i_{3}$. The outputs $o_{i}$ are assumed to be binary, taking values zero or one, which leads to the separable state bounds B≤2. For an ideal GHZ state, however, the quantum bound is *B_q_* = 4. For the Mermin inequality (Eq. 1), there are four measurement settings: $\left\{i_{1},i_{2},i_{3}\right\}=\left\{0,0,1;0,1,0;1,0,0;1,1,1\right\}$ (defined in Eq. 3 in Materials and Methods) each with eight possible outcomes. For a perfect GHZ state, only a subset of outcomes will occur with certainty: We group these together, forming the winning outcomes. To make the inequality into a nonlocal game, one then randomly measures each state of *S* in one of the four settings, records the number of winning events $n_{\text{wins}}$, and computes the experimental winning probability as $P_{\text{exp}}=n_{\text{wins}}/N$.

The intuition is then that, because only states equivalent to the target state can violate the inequality, only they can obtain a $P_{\text{exp}}$ close to 1.

In QSC, the verifier measures $N_{\text{ver}}=\mu N$ states of the initial *N* state ensemble and uses her measurement results to estimate $P_{\text{exp}}$. The remaining (1−μ)N states are then certified. Previous related works were formulated such that only a single copy was certified, i.e., *N*−1 states are measured and only one state is certified (*25*, *26*). By doing so, one can completely remove all IID assumptions. Reference (*21*), instead, formulates QSC for any value of μ, allowing multiple states to be certified. This comes at the cost of still requiring the assumption that the subsequently emitted states are independent but, nevertheless, still removes the identical distribution assumption [see (*21*) for more details]. Given that state independence is a very reasonable assumption for parametric photon-pair sources, here we implement multicopy certification, which may be applicable for a wider class of applications.

To formalize the relation between $P_{\text{exp}}$ and $\bar{\Xi }$ the average extractability, consider a sequence of μN physical states with a reduced average extractability $\bar{\Xi }\leq 1-\eta$. In this case, the maximum winning probability is $P_{\eta }=P_{\text{QM}}-\mathrm{c\eta}$, where *c* is a constant depending on the specific Bell inequality (*21*), and $P_{\text{QM}}$ is the quantum mechanical probability to win the game (which is 1 for our present example). If we then observe $P_{\text{exp}}>P_{\eta }$, the physical states likely have an extractability $\bar{\Xi }>1-\eta$. However, if $P_{\text{exp}}$ is close to $P_{\eta }$, it is possible that finite statistical fluctuations led to an “accidental” win. Thus, we need to know how likely it is that our observation, $P_{\text{exp}}>P_{\eta }$, could be reproduced by a series of states with a fidelity less than F=1−η. This is a classical statistical problem (*44*, *45*). It can be shown that the probability for a false win after μN states are measured is bounded by $\delta \leq e^{-D\left(P_{\text{exp}}∥P_{\eta }\right)\mu N}$, where $D\left(x∥y\right)=x\text{log}\left(x/y\right)+\left(1-x\right)\text{log}\left[\left(1-x\right)/\right.\left.\left(1-y\right)\right]$ is the Kullback-Leibler divergence (*44*). On the basis of this, (*21*) proves that our confidence *C* that remaining (1−μ)N states have an average fidelity greater than 1−η is

$$C\geq 1-\delta =1-e^{-D\left(P_{\text{exp}}∥P_{\eta }\right)\mu N}$$ (2)

which grows to 1 exponentially fast with *N*. See tSupplementary Materials section G for more details.

The scaling of the confidence that our physical states have an extractability larger than 1−η depends on the statistical distance between $P_{\text{exp}}$ and $P_{\eta }$ (Eq. 2). The estimated number of measured states needed to verify this lower bound is $N\geq \frac{\text{ln}\;\delta }{\text{ln}\left[1-\mu +\mu e^{D\left(P_{\text{exp}}∥P_{\eta }\right)}\right]}$ (*21*). Experimentally, instead, we often wish to compute the fidelity from the results of a set of measurement results. We can also use QSC for this. It results in two different scaling behaviors related to the specifics of the Bell inequality used to make the nonlocal game. In particular, not all states have Bell inequalities that achieve a perfect success probability when translated to a nonlocal game. This happens when quantum mechanics cannot achieve the maximum algebraic bound of the Bell inequality. For example, for the CHSH inequality the quantum Tsirelson bound $B_{q}=2\sqrt{2}$ is less than the algebraic bound of 4. When this occurs, we also need to consider the ideal winning probability $P_{\text{QM}}$. These scenarios arise when some winning outcomes do not occur 100% of time. We can still define the set of winning outcomes, but now they are given by the most likely outcomes. When $P_{\text{QM}}<1$, the verifiable infidelity scales as $\eta \propto O\left[\frac{\sqrt{\text{ln}\;\delta^{-1}}}{\mathrm{c\mu}\left(1-\mu \right)\sqrt{N}}\right]$, i.e., with a suboptimal $N^{-1/2}$ scaling. However, when $P_{\text{QM}}=1$, we can obtain the optimal $N^{-1}$ scaling $\eta \propto O\left[\frac{\text{ln}\;\delta^{-1}}{\mathrm{c\mu}\left(1-\mu \right)N}\right]$. Note that these scaling behaviors are derived in the limit of large *N*, and, as we will see later, in our intermediate regime, they are only approximate. Nevertheless, as we will show experimentally, this means that for the two-photon Bell state, using the CHSH inequality, we expect to achieve approximate $N^{-1/2}$ scaling, but the three-photon GHZ state with the Mermin inequality can exceed this scaling.

### Experimental apparatus for QSC

We will now present our experimental implementation of QSC [as proposed by Gočanin *et al.* in (*21*)] for bipartite and tripartite states, allowing us to demonstrate both scaling behaviors discussed above. For the two-photon case, we use a type 0 spontaneous parametric downconversion (SPDC) source in Sagnac configuration (*46*) to generate the Bell state $|\Phi^{+}\rangle =\frac{1}{\sqrt{2}}\left(|\mathrm{HH}\rangle {}_{1,2}+|\mathrm{VV}\rangle {}_{1,2}\right)$, whereas for the tripartite case, we use a three-photon GHZ state $|\Psi_{\text{GHZ}}\rangle =\frac{1}{\sqrt{2}}\left(|\mathrm{HHH}\rangle {}_{1,2,3}+|\mathrm{VVV}\rangle {}_{1,2,3}\right)$ produced by two sandwich-like SPDC sources (*47*). Our three-photon source is a postselected source, meaning that, to ensure that the source has generated the correct entangled state, we must postselect on three photons arriving at each measurement station (as well as detecting an additional trigger photon in the source). With respect to a DI implementation of QSC, this opens up our source to the postselection loophole (*48*–*50*), which cannot be closed with our current methods. Doing so would require a source that directly generates three-photon entanglement (*51*) with high enough coupling efficiency or a heralded three-photon source (*52*, *53*) with sufficiently high heralding efficiency. We stress that this postselection loophole is not related to our use of probabilistic sources, which have been used for other DI protocols (*54*).

For a full description of the photon sources, see Supplementary Materials sections A and B. In both scenarios, we assume that the subsequent states emitted by the source are independent, but we do not need to assume that they are identically distributed.

**Fig. 2.**
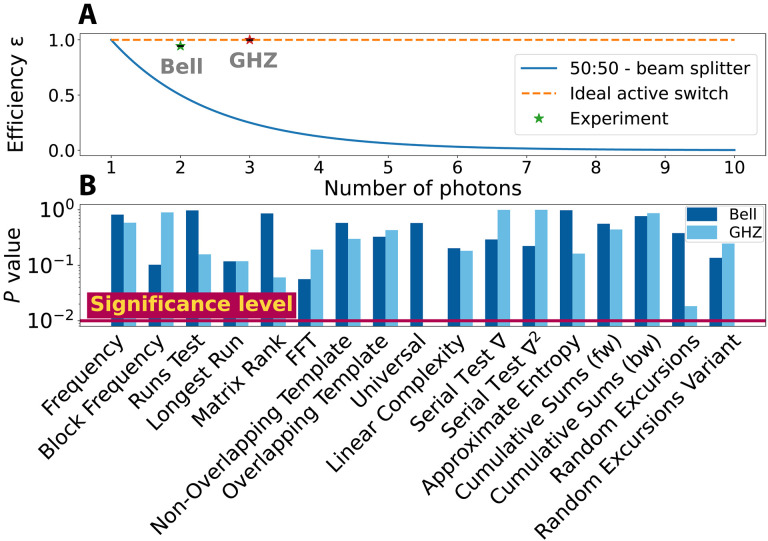
Random sampling characterization. (**A**) Synchronization efficiency. The blue curve plots the maximum synchronization efficiency using probabilistic routing with 50:50 beam splitters versus the number of photons in the state to be certified. The dashed line at 1 indicates the maximum efficiency attainable with the deterministic switches that we use. Our observed two-photon and three-photon efficiencies of $\epsilon_{2}=0.9439\pm 0.0041$ and $\epsilon_{3}=0.9997\pm 0.0066$, respectively, are plotted as stars. (**B**) Results of NIST’s statistical test suite for random number generators applied to our sampling of the produced state for two-photon Bell states in dark blue and three-photon GHZ states in light blue. In both cases, the results exceed the significance threshold of 0.01, indicating that our implemented sampling is consistent with truly random sampling.

To randomly route the multiphoton states between the user and verifier for QSC, one could use passive beam splitters. Although this would work perfectly for a single photon state, it introduces significant loss for *M*-photon states. For QSC, we need all of the photons to either arrive at the user or verifier; situations where, for example, one photon arrives at the user and another at the verifier count as loss events. Because, with 50:50 beam splitters, each photon is independently routed, all *M* photons will arrive at either the user or verifier with probability $\epsilon ={\left(\frac{1}{2}\right)}^{M}$. In other words, the photons are not synchronously routed between the user and verifier. This is the passive synchronization efficiency plotted as the blue line in Fig. 2A. In a fully DI implementation of QSC, the detection efficiency must remain sufficiently high. This synchronization loss serves to decrease the detection efficiency, making DI-QSC impossible with passive beam splitters. Even if a DI implementation is not desired, the loss introduced by sampling with passive elements tends to 0 exponentially fast, making active elements essential for larger entangled states.

To circumvent this loss, we use *M* synchronized OSs (see Fig. 1). Because of the different photon production rates, we use different technologies for our two-photon and three-photon experiments, explained in Materials and Methods (“Deterministic random sampling with OSs”). In any case, in both experiments, all *M* OSs switch between the user and verifier. Both switches are driven with a fixed 50% duty cycle to achieve μ≈0.5. Experimentally, the duty cycle is set electronically via the periodic function generator that drives the OSs: The duty cycle of the function generator is nominally the duty cycle of the OSs and thus directly yields the sampling rate. Thus, although we only study μ≈0.5, it can be easily tuned to implement other sampling rates. Note that changing the duty cycle does not significantly change the synchronization of the OSs. We show below how this can still be used to randomly sample from the states. To characterize the QSC efficiency for both experiments and show that we exceed the passive synchronization efficiency, we measure the two and three rates at the user’s (verifier’s) measurement station $R_{\text{use}}$ ($R_{\text{ver}}$), as well as all two-photon and three-photon events between the two measurement stations $R_{\text{cross}}$ (nominally, $R_{\text{cross}}=0$). Then, the experimental synchronization efficiency is $\epsilon =\left(R_{\text{use}}+R_{\text{ver}}\right)/\left(R_{\text{use}}+R_{\text{ver}}+R_{\text{cross}}\right)$. These are the data points shown in Fig. 2A, confirming that we have routed the photons with high efficiency. See Supplementary Materials section C for more details. Although the total loss in our experiment is too high to close the detection loophole, our synchronization efficiency is high enough (0.9439 ± 0.0041 and 0.9997 ± 0.0066 for our two-photon and three-photon states, respectively) that it would not significantly affect the detection loophole in an otherwise loophole-free experiment. In Materials and Methods (“Loophole analysis”), we show that also including the insertion loss of the switches used in our two-photon experiments would decrease the overall efficiency too much to remain loophole-free. Although the switches used in our three-photon experiment are already sufficient in efficiency, the relatively slow speed would make the freedom of choice loophole hard to close.

Our OSs sequentially alternate between the user and verifier, which, on its own, does not implement random sampling. To that end, we make use of SPDC’s inherent randomness (making a device-dependent assumption). In particular, we drive our OSs much faster than our sources produce photons the rate of producing photons. Thus, when a given multiphoton state is randomly emitted, the OSs will randomly be in one configuration or the other. To confirm this, we call an *M*-photon detection at the user (verifier) a “0” (“1”) and generate a bit string. We test these bit strings with NIST’s statistical test suite for random number generators (*55*). As elaborated on in Supplementary Materials section D, we obtain an overall confidence of 0.99 that our produced bit strings are random. Figure 2B shows the results of the individual tests. Although this analysis indicates that we have achieved random sampling, doing so requires us to make a device-dependent assumption that the randomness originates from the source. For a fully DI implementation, the switches should be driven randomly, as we discuss in Materials and Methods (“Loophole analysis”).

Once the photons arrive at the verifier’s measurement station, she must randomly choose a measurement setting. For our two-photon and three-photon states, we use the measurement sets $M_{\text{CHSH}}$ and $M_{\text{Mermin}}$, respectively, which are defined in Eq. 3 in the Materials and Methods section. In both cases, the measurements are implemented with standard methods (waveplates and polarizing beam splitters), but because of the different photon production rates, we randomly sample from the measurements differently (see Fig. 1, B and C).

### Experimental results

With our QSC apparatus in place, we drive our OSs with 50% duty cycle pulses so that the verifier takes approximately half of the photons for her measurements. In our two-photon measurements, this results in counting rates of $R_{\text{user}}∼33$ kHz ($R_{\text{ver}}∼25$ kHz) at the user (verifier), yielding a sampling probability of $\mu_{\text{Bell}}\approx 0.43$ for our two-photon experiment. For the three-photon experiment, we have $R_{\text{user}}∼0.3$ Hz ($R_{\text{ver}}∼0.25$ Hz) at the user (verifier), yielding $\mu_{\text{GHZ}}\approx 0.45$. Note that we have defined $\mu =R_{\text{ver}}/\left(R_{\text{user}}+R_{\text{ver}}\right)$. As mentioned above, QSC can be performed for any value of μ. Here, we set it close to 0.5 so that the user and verifier measure approximately the same number of measurement results to ease their comparison.

**Fig. 3.**
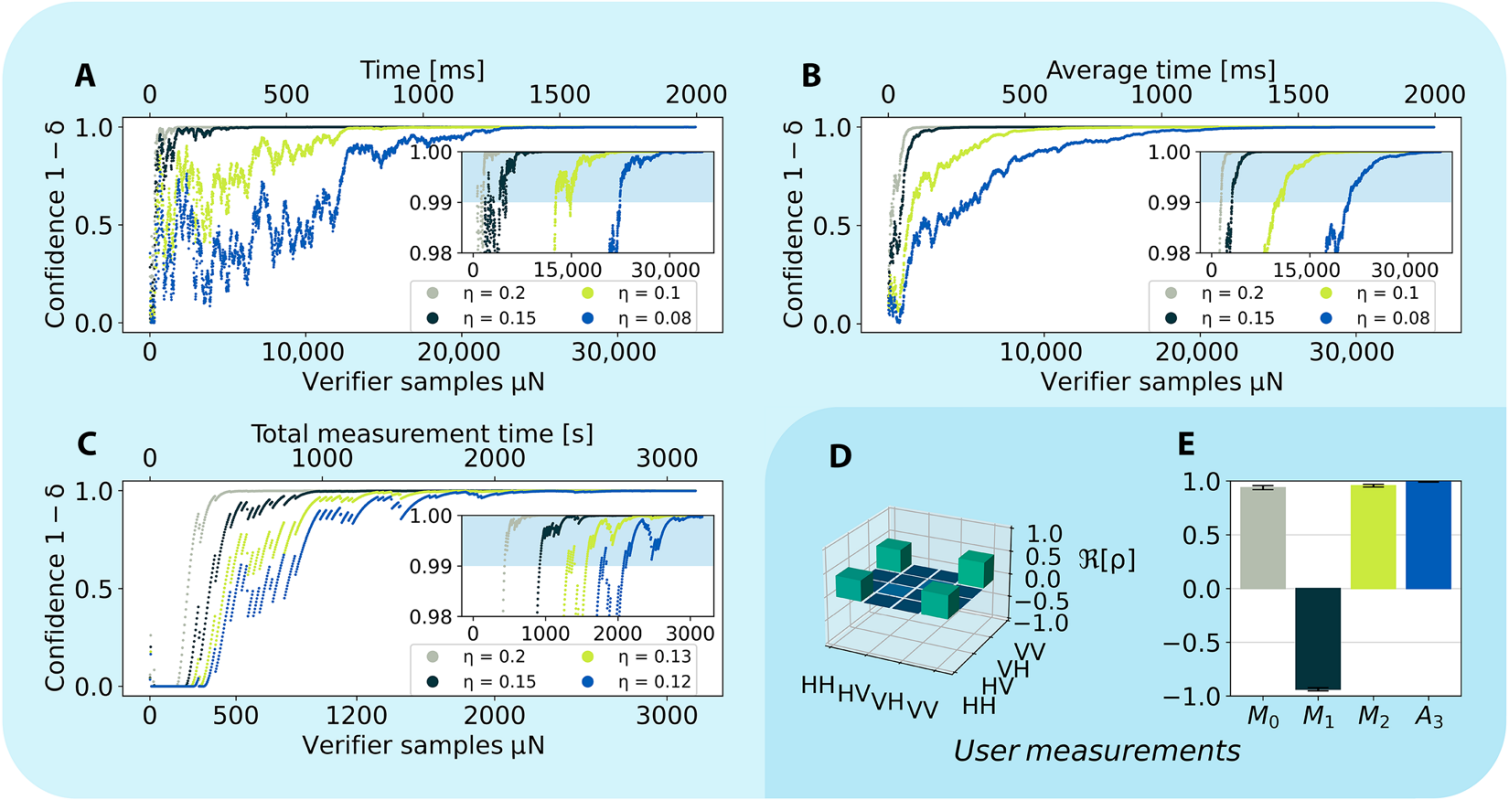
Confidence in two- and three-photon QSC. (**A**) Confidence versus the number of verifier measurements (bottom axis) and measurement time (top axis). The same dataset is analyzed for different fidelity F=1−η lower bounds. Less stringent bounds (higher values of η) converge to a confidence of 1 more quickly. The inset shows the high-confidence region, with the shaded area representing a confidence above 0.99. (**B**) The two-photon measurement presented in (A) is repeated 12 times and averaged. The data are plotted as in (a). (**C**) Verifier’s confidence growth for our three-photon GHZ states, plotted as in (a). (**D**) Real part of the density matrix of our two-photon states measured with standard quantum state tomography, taken by the user concurrently with the verifier’s measurements. The plotted density matrix has a fidelity of 0.9947 ± 0.0002 with the target $|\phi^{+}\rangle$ Bell state. (**E**) Results of the user’s GHZ witness measurement on our three-photon states, yielding a fidelity of 0.9678 ± 0.0055 with the target GHZ state. The labels on the *x* axis refer to specific measurement settings, defined in the Supplementary Materials.

Although the verifier implements QSV, the user operates his measurements independently. We implement two different characterizations for the user and check their consistency with the verifier. First, the user implements standard device-dependent characterizations. For our two-photon source, the user performs full quantum state tomography, finding a fidelity of 0.9947 ± 0.0002 with the target Bell state (see Fig. 3D). Given the lower three-photon count rate and the larger number of required measurements, for three photons, the user instead uses a GHZ witness (*56*, *57*) to estimate a fidelity of 0.9678 ± 0.0055 (see Fig. 3E). We also perform standard DI self-testing with the user’s measurement device, finding a fidelity of 0.971 ± 0.005 and 0.9032 ± 0.0066, for our two-photon and three-photon states, respectively. See Supplementary Materials section F for more details. The discrepancy between the device-dependent and DI techniques is well known, arising because it is, in general, easier to place tighter bounds in a device-dependent scenario (*58*).

Although the user performs his measurements, the verifier implements QSV on her subset. To do so, she simply records the number of winning and losing events and computes the winning probability $P_{\text{exp}}$ as a function of the number of measurements. $P_{\text{exp}}$ for both bipartite and tripartite scenarios is provided in Supplementary Materials section E. To use $P_{\text{exp}}$ to certify the physical states, the verifier sets a minimum fidelity 1 − η and uses Eq. 2 to compute her confidence in that lower bound. In Fig. 3A, we plot this confidence versus the number of measurements made by the verifier μ*N* for a range of infidelities from η = 0.08 to η = 0.2 for the two-photon states. Figure 3C shows the same data for our three-photon states for a range of η = 0.12 to η = 0.2. For each dataset, we observe a series of rapid increases and sharp drops. As μ*N* increases, the drops become less pronounced and the confidence tends to 1. The periods of increasing confidence are caused by successive winning events, whereas the drops are caused by losses.

We also stress the measurement times for these datasets. In the two-photon data, in less than ≈1 s, the verifier reports a fidelity above 0.9 with confidence ≫ 0.99, demonstrating the applicability of our methods. Given our lower three-photon rates, we achieve similar values in ≈30 min for the GHZ states. Nevertheless, in both scenarios, the user and verifier can operate concurrently, with only a constant decrease in the user’s count rate.

The effect of changing μ is apparent from the above discussion. Because the user and verifier act completely independently, changing μ effectively only changes the total elapsed time that it takes for the verifier’s measurements to converge, i.e., it will only change the upper time axis in Fig. 3 (A to C). This is because the confidence scales with μ*N*. Thus, μ may be set differently for different scenarios. For example, if it is important that the user maintains the highest possible count rate, μ can be made very small. In this case, it will take the verifier longer to converge, but if the total runtime of the experiment is sufficiently large, this may not be an issue. On the other hand, if one wishes to issue a certificate quickly, then it may be advantageous to increase μ, at a cost that the user will receive a lower rate. Because, in the present work, we are primarily interested in showing that the user and verifier’s predictions are consistent, we chose a balanced value of μ ≈ 0.5.

To smooth out the statistical fluctuations in the two-photon data, we perform 12 rounds of QSV with 35,000 samples and average the results. This results in the curves in Fig. 3B. We identify the minimal sample number required to reach 99% confidence level. The insets in Fig. 3 (A and B) present zoomed-in plots of the high-confidence region, and the blue-shaded areas denote the area above 99%. From the inset in Fig. 3B, we can estimate the number of samples need to certify infidelities below η = {0.2,0.15,0.1,0.08}, finding that {1420,3106,10019,20982} samples are needed, respectively, for our two-photon Bell states.

The main difference between the two-photon and three-photon datasets is related to the different values of $P_{\text{QM}}$. For our two-photon CHSH results, $P_{\text{QM}}=\frac{2+\sqrt{2}}{4}\approx 0.85$. For the Mermin inequality, however, $P_{\text{QM}}=1$, leading to fewer failed events and thus smoother data in a single experimental run, obviating the need to average to estimate the required number of measurements. Overall, for the three-photon experiment, we have a success probability $P_{\text{exp}}∼0.97$, which leads to {433, 919, 1562, 2111} samples to certify infidelities η = {0.2, 0.15, 0.13, 0.12} with 99% confidence, respectively.

**Fig. 4.**
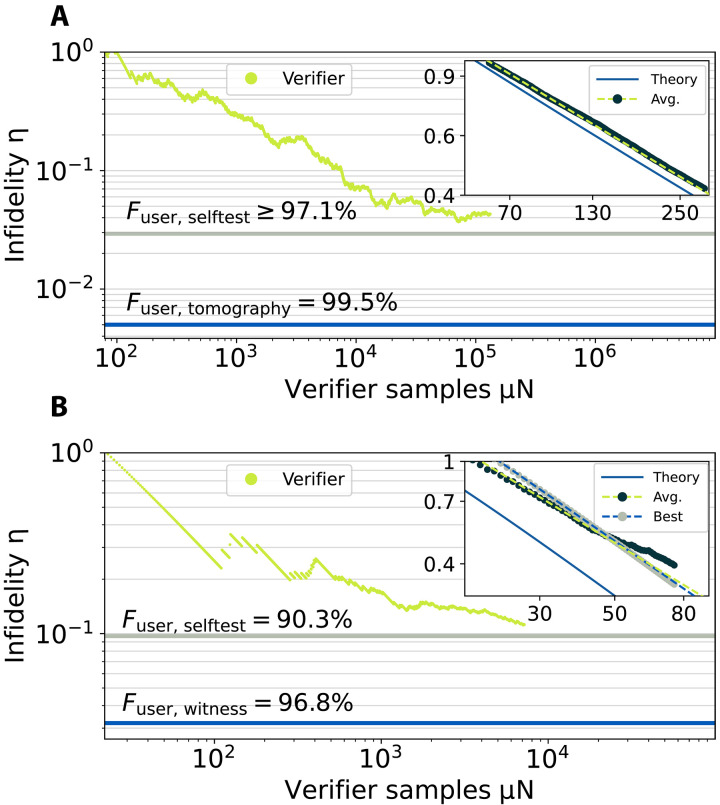
QSC fidelity scaling at 99% confidence. The estimated infidelities η when setting the confidence *C* = 1 − δ = 0.99 for (**A**) two-photon Bell states and (**B**) three-photon GHZ states plotted versus the verifier’s measurement number. In both cases, the infidelity η certified by the verifier (green points) asymptotically approaches infidelity estimated by the user with self-testing (gray lines). The user estimates lower infidelities when using device-dependent methods (blue lines). Insets: The insets plot averaged results over smaller sample ranges to estimate the scaling *s*. The dark points are the averaged data, and the green dashed lines are linear fits with slopes of $s_{\text{Bell},\text{Avg}.}=-0.549$ and $s_{\text{GHZ},\text{Avg}.}=-0.801$, respectively. The blue solid line is theory, demonstrating scalings of $s_{\text{Bell},\text{Theory}}=-0.566$ and $s_{\text{GHZandTheory}}=-0.935$. In the inset of (B), we additionally plot the best-case scaling from one data run that achieves the scaling of $s_{\text{GHZ},\text{Best}}=-0.907$.

As discussed above, different values of $P_{\text{QM}}$ lead to different scaling behaviors for our two-photon and three-photon measurements. To observe this, we again perform QSC, now directly comparing the verifier’s claims on the infidelity to that measured by the user. For the verifier, we then instead fix the confidence level 1 − δ to 0.99 and numerically solve Eq. 2 for the infidelity η. This then represents the highest DI infidelity that our sequence of measurements is consistent with at a 99% confidence level. In other words, it establishes a 99% confidence lower bound on the fidelity of the experimentally generated state. On the basis of this, we plot η versus the number of verifier measurements μ*N* in Fig. 4. Therein, Fig. 4A shows the two-photon data, and Fig. 4B shows the three-photon data. The bounds in both plots represent two alternative methods to estimate the fidelity discussed above. The bounds indicated by the blue lines come from device-dependent measurements [quantum state tomography for the two-photon case and GHZ witness (*56*, *57*) for our three-photon states]. On the other hand, the bounds in gray come from DI self-testing (Supplementary Materials section F). All of these bounds are measured by the user. In both cases, when sufficient data are acquired, QSC converges to the DI self-testing bound. These data show that our QSC protocol is consistent: The infidelities the user measures (with DD or DI methods) are all lower than the infidelities reported by the verifier.

Last, we analyze the scaling of the infidelity as a function μ*N*. To this end, we average several repetitions of the experiments over a fixed sample range. The resulting averaged estimated infidelities are shown in the insets of Fig. 4. For the bipartite case, we take 1398 repetitions for *N* from 1 to 300 (points in Fig. 4A, inset), performing a linear fit in the log-log scale and obtaining a slope of −0.549 (green dashed line in the same inset). This means that the scaling is $\eta \propto N^{-0.549}$. This already exceeds the predicted asymptotic scaling of $N^{-0.5}$. Although this may seem unexpected, both the Heisenberg limit optimal $N^{-1}$ scaling and the suboptimal $N^{-1/2}$ scaling are derived asymptotically and cannot be expected to be literally the same for finite statistics. We numerically predict the expected scaling for our parameter regime by substituting $P_{\text{exp}}=P_{\text{QM}}\approx 0.85$ into Eq. 2 and solving for η as a function of *N*. This is plotted as the blue solid line, which has a slope of −0.566. This agrees quite well with our experimentally measured scaling. For the tripartite case, we use 20 repetitions for *N* from 1 to 75 (dark points in Fig. 4B, inset), and fitting to these data gives a scaling of $\eta \propto N^{-0.801}$ (green dashed line Fig. 4A, inset), which significantly exceeds the suboptimal scaling of $N^{-1/2}$.

Notice that, in both cases (main plots in Fig. 4, A and B), the scaling behavior of the infidelity at larger μN deviates from the ideal case, i.e., it is not linear. This is the correct behavior because our experimental states are not perfect. Therefore, the infidelity should not decrease indefinitely, but it should begin to level off as sufficient data are acquired. To assess the scaling in spite of this, we select a data range from Fig. 4B for small values of μ*N* where all the samples pass the nonlocal game {from N∈[10,100]} (gray points in the same inset) and fit the result (blue dashed line). Doing so yields a scaling of $\eta \propto N^{-0.907}$. We compare this to the numerically predicted scaling behavior for ideal GHZ states as before: We use Eq. 2 with $P_{\text{exp}}=1$ and obtain $\eta \propto N^{-0.935}$ (blue line). This again agrees well with our experimentally measured scaling, with a small discrepancy coming from failed events before the displayed range. Both our two-photon and three-photon data qualitatively show the expected trends.

## DISCUSSION

**Fig. 5.**
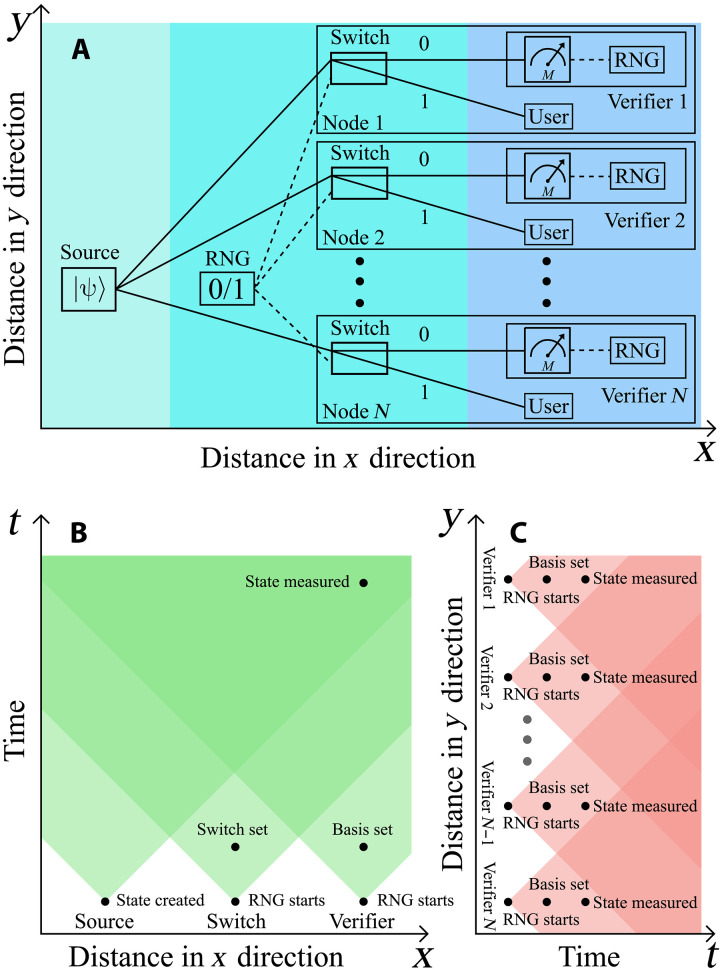
Proposal for DI-QSC. (**A**) A heralded *N*-photon source sends photons to *N* space-like separated nodes. An RNG also distributes signals to the *N* nodes, which are used to synchronize trusted switches in each node. In addition to these switches, each node hosts a user and verifier measurement station. The verifier measurement stations operate device independently. (**B** and **C**) Spacetime diagrams along the *x* and *y* directions, respectively. In Materials and Methods (“Loophole analysis”), a loophole analysis of this setup is presented.

We have presented an experimentally feasible method for QSC. To do so, we used active switches to deterministically implement a random sampling of multiphoton states from two-photon and three-photon sources. A verifier measures the sampled states, issuing a statistically rigorous certificate to the user that the remaining states have average fidelity to a target state above some threshold. From the user’s point of view, the only noticeable effect of the verifier is a slight reduction in the counting rate that does not scale with the number of photons in the entangled state. Moreover, we have demonstrated two distinct scaling behaviors related to different features of nonlocal games, i.e., the winning probability. Although we have shown this technique for Bell states and GHZ states, it can be generalized to any states for which a robust self-testing protocol exists. We also mention that, although the theory underlying our work is DI, our implementation contains loopholes and is thus not DI. However, to guide future DI implementations, Fig. 5 presents a potential extension of our experiment, which could close the loopholes needed to claim device independence. See Materials and Methods (“Loophole analysis”) for a full description of this proposal.

To summarize, we have performed QSC (as opposed to verification), showing how to use active switches randomly sample a subset of the available states to certify the remaining states. We have demonstrated the efficiency of QSC, experimentally beating the standard limit of $N^{-0.5}$ with $N^{-0.801}$. Moreover, QSC removes the assumption of identically distributed states and can be implemented in a DI manner. Although our experiment is not DI, we have proposed how this could be achieved in future work. Last, given the relative ease of our realization, requiring only local measurements and low postprocessing complexity, it would be interesting to tailor QSC for other quantum systems, such as trapped ions (*59*, *60*), cold atoms (*61*, *62*), and superconducting circuits (*63*). We thus anticipate that our work could be used to benchmark future large-scale quantum devices.

After the completion of our experiment, we became aware of a closely related work (*64*).

## MATERIALS AND METHODS

### Deterministic random sampling with OSs

As discussed in the main text, we use OSs to redirect photons to the verifier station. For both our two-photon and three-photon experiments, we use fiber-coupled OSs. For our two-photon experiment, the switches are based on integrated electro-optic modulators (EOMs), whereas our three-photon experiments use microelectromechanical system (MEMS)–based switches. For our two-photon work, we use one Agiltron 2x2 Nanospeed switch and one BATi Inc. 2x2 Nanona fiber switch. These switches feature rise and fall times of ~100 and 50 ns, respectively. Both devices have a maximum repetition rate of 1 MHz and exhibit a cross-talk between output ports of around 20 dB. The transmission of each switch is ≈79%, resulting in a two-photon transmission of 0.79^2^ ≈ 0.62. In the three-photon experiment, we use three identical Agiltron 1x2 fiber optical MEMS switches, each with a rise and fall time of ~2 ms, a maximum repetition rate of 5 Hz, and a cross-talk between output ports ranging from 50 to 75 dB. The transmission of each switch is ≈95%, resulting in a three-photon transmission of 0.95^3^ ≈ 0.85. In both cases, we synchronize the OSs and set them to alternate between the user and verifier stations with a nominal 50% duty cycle.

To ensure that states randomly directed to the verifier, there are two essential conditions need to be satisfied: (i) The verifier (or user) should receive the full copy. For example, for the three-photon experiment, this means that all three photons should be sent to the same measurement station. To this end, we synchronize the OSs with an external trigger signal. This is verified in Fig. 2A and in Supplementary Materials section C. The discrepancy in Fig. 2A between the ideal switching efficiency (ϵ = 1) is due to the “synchronization efficiency,” as discussed in the main text, and this can be reduced by nonzero cross-talk between switch output ports. The EOM-based switches used in the two-photon experiment exhibit higher cross-talk than the MEMS-based switches, leading to a nonzero probability of single photons ending up in the “wrong” output port, thereby reducing ϵ. (ii) Distribution of each copy is random. Although we drive the switches with a deterministic signal, we use the intrinsic randomness from the SPDC process, wherein each photon pair is generated at a random time. Therefore, when each state is generated, it can randomly lie in a time slot that will distribute it to the verifier or user. However, this requires the *M*-photon generation rate to be substantially lower than the switching rate to avoid having one of more copies of the state in each time slot. Consequently, we set our switches speeds to 800-kHz and 5-Hz repetition rates for the two-photon Bell states and three-photon GHZ states, respectively. In both cases, this is significantly higher than the respective state generation rates of ≈62 and ≈0.5 Hz. Figure 2 verifies the validity of this approach.

### Measurement strategy

Our DI-QSC protocol is based on converting the CHSH and Mermin inequalities into two-photon and three-photon nonlocal games, respectively. This works by considering the different observables used to violate the respective inequalities and associating each observable with a binary classical input ascribed to each local party. In particular, for the bipartite case, we have the possible inputs $\left(i_{1},i_{2}\right)=\left\{(0,0),(0,1),(1,0),(1,1)\right\}$, and for the tripartite case, we have $\left(i_{1},i_{2},i_{3}\right)=\left\{(0, 0, 1),(0, 1, 0),(1, 0, 0),(1, 1, 1)\right\}$. These inputs then refer to the following observables

$$\begin{array}{c} M_{\text{CHSH}}=\left\{X_{1}⊗\frac{X_{2}+Z_{2}}{\sqrt{2}},X_{1}⊗\frac{X_{2}-Z_{2}}{\sqrt{2}},Z_{1}⊗\frac{X_{2}+Z_{2}}{\sqrt{2}},Z_{1}⊗\frac{X_{2}-Z_{2}}{\sqrt{2}}\right\}\\ M_{\text{Mermin}}=\left\{Y_{1}⊗Y_{2}⊗X_{3},Y_{1}⊗X_{2}⊗Y_{3},X_{1}⊗Y_{2}⊗Y_{3},X_{1}⊗X_{2}⊗X_{3}\right\} \end{array}$$ (3)

In both cases, the result is four different multipartite measurements that must be made randomly.

To implement the random selection of measurements for our two-photon experiment, we use a 50:50 beam splitter in the path of each photon to direct that photon to physically different measurement apparatus corresponding to the binary inputs (Fig. 1B). This enables us to randomly select our measurements at very high counting rates, but it comes at the cost of opening the freedom-of-choice loophole. Nevertheless, because our measurement stations are not space-like separated, our experiment is open to the locality loophole. Thus, because our experiment is, in any case, open to loopholes, we chose the measurement bases passively for simplicity. Because our three-photon rate is significantly lower, we instead only have a measurement device per photon. The random classical inputs are then generated by a commercial quantum random number generator (QRNG), the Quantis QRNG USB from ID Quantique. These outputs of this QRNG are set to trigger motorized waveplates that change the measurement settings. To further ensure that our sampling is independent, we switch the settings every second (which is faster than the generation rate) and only analyze measurements time slots that contain no more than one three-photon event.

### Loophole analysis

#### Standard loopholes

DI protocol implementations ensure robust protection against any external manipulation or influence on the protocol, maintaining reliable and trustworthy outcomes. In principle, even if the entire apparatus were controlled by an untrusted party, the protocol would still deliver correct results. Implementing DI-QSC requires a loophole-free violation of a Bell inequality (the CHSH or Mermin inequality in our case), and thus the common loopholes—i.e., the (1) locality, (2) freedom of choice, and (3) detection—must be closed (*49*). We do not achieve this in our work. Although loophole-free Bell tests have recently been conducted (*65*–*67*), such demonstrations are rare due to the stringent infrastructure requirements involved. Nevertheless, in Fig. 5A, we sketch an *N*-partite extension of our experiment that could address these three loopholes, as well additional ones related to multipartite QSC. To close the locality loophole (1), a source at a central location generates the *N* entangled photons, which are distributed to *N* space-like separated nodes. The measurement basis must then be set after the state are generated, which can, in principle, be easily enforced (Fig. 5, B and C). To address the freedom-of-choice loophole (2), the measurement basis must be randomly set. In our three-photon experiment, we satisfy this by implementing our basis choice using a QRNG. In our two-photon experiment, the basis is set randomly using 50:50 beam splitters. Although this is a random process, it opens the loophole because the photons could carry hidden variables determining whether they are transmitted or reflected to one measurement setting or the other. Last, to ensure that the detection loophole (3) is closed, the overall transmission between the source and the detectors (including the detection efficiency) must remain over a certain threshold. This is 66.7 and 75% for the two-photon and three-photon experiments, respectively (*68*).

#### Preparation postselection loophole

Although the above loopholes apply to all Bell tests, current methods of creating multipartite entangled states open up an additional loophole, which we refer to as (4) the preparation postselection loophole. This loophole is one of the primary challenges for a loophole-free multipartite demonstration of quantum nonlocality, which has not yet been realized (*50*). Closing this loophole is required for DI-QSC of multiphoton states. The preparation postselection loophole comes from the fact that we use nonheralded gates to build three-photon entanglement from two-photon entangled states. We can only be sure that we have successfully generated the entangled state when one photon is detected in each output port [see (*50*) for a discussion of the implications]. To close the postselection loophole, one could use heralded sources of multiphoton entanglement, as in (*53*), which can be achieved with either deterministic sources or nondeterministic SPDC as we use here. Unfortunately, the heralding efficiency in such experiments is still relatively low and thus opens the detection loophole.

#### Loopholes specific to DI-QSC

As above, we have discussed loopholes only related to loophole-free violations of Bell inequalities, which must also be closed for DI-QSC. However, DI-QSC brings additional loopholes. Here, we identify and discuss two additional loopholes: (5) the switching-loss loophole and (6) the random sampling loophole. Note that we also assume that the switches belong to the measurement nodes and are thus trusted devices. We will now discuss how our proposal in Fig. 5 addresses these loopholes.

We already alluded to the switching-loss loophole in the main text. The intuition is relatively straightforward: Upon insertion of the switches used to implement the random sampling, the transmission of the *N*-photon state from the source to the verifiers should remain above the threshold needed to close the detection efficiency loophole. There are two contributions that reduce this transmission. The first is simply the transmission loss of the switches. In our case, for the two-photon experiment the transmission is ≈0.79 per switch, whereas for the three-photon experiment, it is ≈0.95 per switch. This results in an overall two-photon transmission of 0.79^2^ = 0.62 and a three-photon transmission of 0.95^3^ = 0.85. Thus, although our two-photon switches are too lossy, our three-photon switches could, from a loss perspective alone, meet the requirements to close the loophole.

The other source of loss is the synchronization efficiency, already discussed in the main text and in Materials and Methods (“Deterministic random sampling with optical switches”). For completeness, we discuss it here in the context of loopholes. When an *N*-photon state is selected to be sent to the verifier, all *N* photons must simultaneous be routed. If some photons are sent to the user and others in the verifier, these events introduce loss. If this loss reduces the source-to-verifier transmission too much, the detection loophole is opened. This is the reason that we use synchronized active switches. If one uses “passive switches” (i.e., beam splitters), this loss scales to zero exponentially fast with the number of photons in the entangled state. We call this the passive synchronization efficiency and plot it for the case of 50:50 beam splitters in Fig. 2A. Using synchronized active switches, on the other hand, can, in principle, route the photons without introducing any additional loss. Experimentally, we achieve synchronization efficiencies of 0.9439 ± 0.0041% and 0.9997 ± 0.0066% for our two-photon and three-photon experiments, respectively, which could be sufficiently high to remain above the detection efficiency threshold if other experimental losses were low enough.

The final loophole we discuss is the random sampling loophole (6). If the sampling process is not genuinely random, the validity of the protocol is compromised. The challenge in enforcing this experimentally is that the switches, which are located in space-like separated nodes, must be synchronized and the decision of whether to route the *N*-photon state to the verifier or to the user must be made after the source produces a state, just as is done in choosing the basis to close the standard freedom-of-choice loophole. Nevertheless, this can be enforced with space-like separation. The switch rise time must also be considered. In our experiment, the three-photon switches exhibit a rise time of 2 ms, corresponding to a fiber delay of several hundred kilometers, which introduces substantial loss. Therefore, OSs suitable for a fully DI implementation must combine low loss with sufficiently high switching speed (*69*, *70*).

#### A loophole-free proposal

To show how our proposed setup can achieve close these six loopholes, we will now analyze each component shown in Fig. 5A, moving from left to right. Corresponding spacetime diagrams for the *x* and *y* directions are presented in Fig. 5 (B and C), respectively. We first assume an ideal photon source capable of generating a heralded multiphoton state, i.e., one that closes loophole 4.

The next element in Fig. 5 is a random number generator, which produces a random binary output (“0” or “1”) to determine whether the OSs will direct the state to the verifier or the user. It is essential that the random number generator operates in a manner such that it selects the user or verifier before the photon source can signal to it (closing the loophole 6), and it can signal to the switches before each of the *N* photons can arrive at their respective node (so the switches can be synchronized and close loophole 5). Note that, in our experiment, we use randomness in the photon generation time to implement the random sampling, which, although random, does not close this loophole. Achieving this requires aligning the space-like separation with the speed and timing of the electronics involved. It may also be possible to close this loophole by making the appropriate shielding assumptions as done in recent DI experiments (*71*, *72*).

Once the switches are configured, two scenarios arise. If the state is routed to the user, no loopholes need to be addressed (unless the user also wishes to operate device independently, but we will not focus on that here). Conversely, if the states are directed to the verifier, device independence is necessary, i.e., loopholes 1 to 3 must be closed. Doing so requires the *N* verifiers to select their measurements randomly independently and without correlation to the photon source. To ensure freedom of choice, the measurement settings must be determined before any signal from the source can influence a verifier. In photonics, this is typically achieved using an RNG in combination with a fast Pockels cell.

In addition, all *N* verifiers must be space-like separated from each other to prevent any causal influence in their measurements. This requirement is represented in the spacetime diagram for the *y* direction in Fig. 5C. Last, addressing the detection loophole necessitates achieving a system detection efficiency above a specific threshold. To close this loophole, the setup must use low-loss optical components, high-efficiency detectors, and sufficiently high coupling efficiencies between free space and fiber. Overall, implementing DI-QSC is a challenging goal, but no more challenging than implementing most other multipartite DI protocols.

## Supplementary Materials

### This PDF file includes:

Supplementary Text Figs. S1 to S5 References
