# Supplementary material for: Experimental quantum state certification by actively sampling photonic entangled states
Source: Sci Adv. 2026 Feb 11;12(7):eaea4144. doi: 10.1126/sciadv.aea4144 (PMC12893295; doi:10.1126/sciadv.aea4144)
Supplement: Supplementary file 1 — Supplementary Text Figs. S1 to S5 References [file sciadv.aea4144_sm.pdf]

Science Advances
AAAS

Supplementary Materials for

Experimental quantum state certification by actively sampling photonic entangled states

Michael Antesberger *et al.*

Corresponding author: Huan Cao, huan.cao@univie.ac.at; Philip Walther, philip.walther@univie.ac.at

Sci. Adv. **12**, eaea4144 (2026)
DOI: 10.1126/sciadv.aea4144

This PDF file includes:

Supplementary Text
Figs. S1 to S5
References

A. Two-Photon Source

For our two-photon experiment we use a type-0 spontaneous parametric down-conversion (SPDC) source as in Ref. [46], illustrated in Fig. S1. The source uses a periodically-poled Lithium Niobate (ppLN) crystal that is placed in a Sagnac loop and pumped by a narrowband continuous-wave laser at 775.06 nm. Before the Sagnac loop, a half waveplate (HWP) sets the pump polarization to 45°. After the entangled photon pairs are created, they pass through a long pass filter (LPF) in order to suppress the remaining pump light. The entangled photon pairs are then seperated with a 100 GHz dense wavelength division multiplexing (DWDM) module. Due to energy conservation in the SPDC process, entangled photon pairs are separated in DWDM channels symmetric around the center of the SPDC emission spectrum, which is at 1550.12 nm. In this experiment we use entangled pairs from DWDM channels 32 and 36, with center wavelength 1551.72 nm and 1548.51 nm, respectively. Each photon is sent to an individual optical switch (OS), which are synchronized with each other to route photon pairs either to the verifier or the user. At the verifier, each photon passes a 50:50 BS to ensure randomness in the choice of the measurement basis. Depending on whether the photon is transmitted or reflected, a different measurement is performed. At the user side, the measurement settings can be set to arbitrary basis using waveplates.

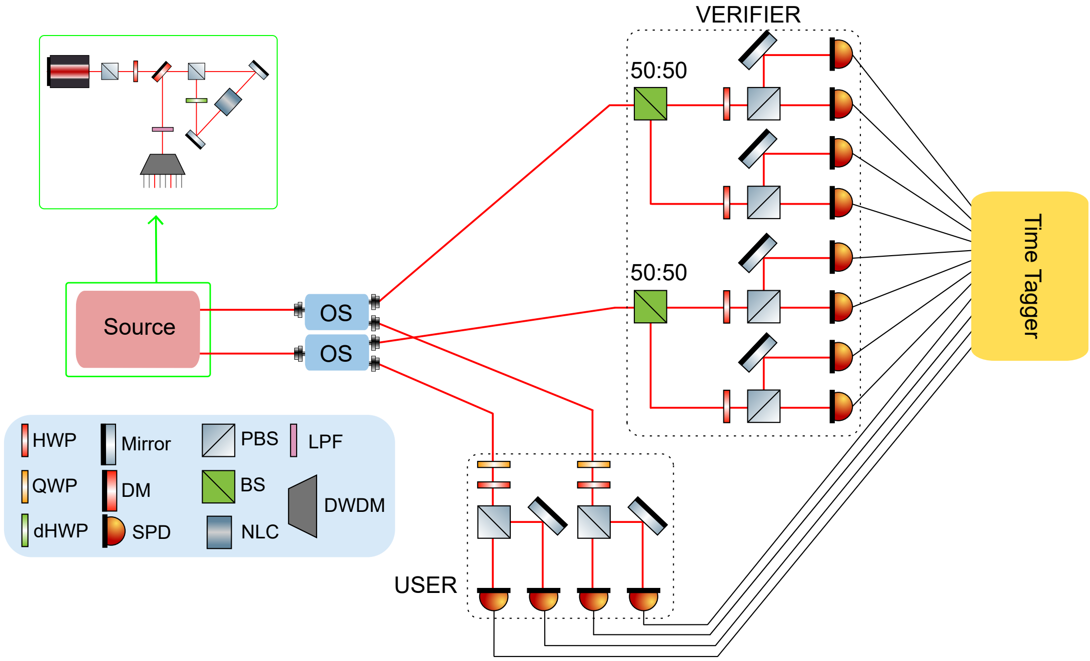

Figure S1: **Two-Photon Experimental Setup.** A continuous-wave laser at 775.06 nm pumps the nonlinear crystal (NLC) to create entangled photon pairs in the telecom C-band centered around 1550.12 nm via type-0 SPDC (spontaneous parametric down-conversion). The DWDM divides the total photon spectrum into several channels. The entangled photons are found in channels equidistant from the center of the spectrum. Each photon is forwarded to one of the two optical switches that are driven by synchronized square signals with 50% duty cycle. This means that approximately half of the photon pairs are sent to the verifier and the other half to the user. The photons sent to the verifier are split at a 50:50 BS, in order to randomly choose the measurement settings. Coincidence detection is performed using a time tagger. The photons at the user side can be used for some other purpose. In our experiment, they are measured with waveplates and polarizing beamsplitters. Abbreviations: HWP, half-wave plate; QWP, quarter-wave plate; dQWP, dual-wavelength quarter-wave plate; DM, dichroic mirror; SPD, single photon detector; PBS, polarizing beam splitter; BS, beam splitter; LPF, longpass filter; DWDM, dense wavelength division multiplexer; OS, optical switch;

B. Three-Photon Source

A sketch of our three-photon experiment is shown in Fig. S2. It consists of two sandwich-like entanglement sources [47]. Each sandwich-like entanglement source consists of two type-II BBO crystals with a true-zero-order half-wave plate (THWP) in between. When pumped by a laser, the first BBO produces two photons in the state $|H\rangle_e|V\rangle_o$,

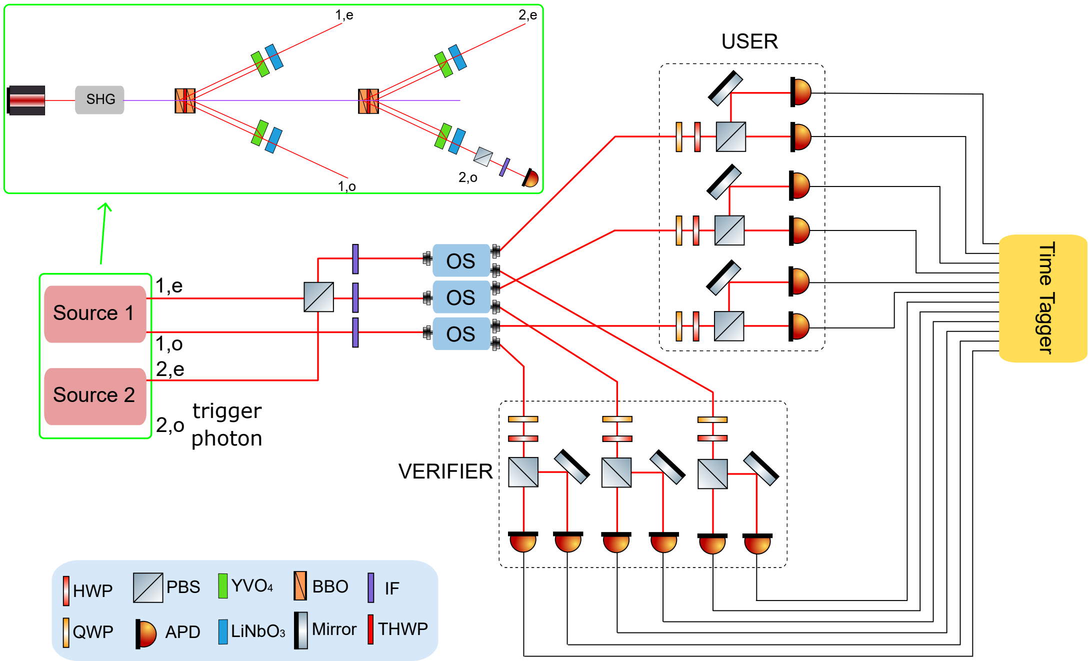

Figure S2: **Three-Photon Experimental Setup.** The two sources are pumped with a pulsed beam at a wavelength of 390 nm, produced from second-harmonic generation (SHG) of a Ti:Sapph laser. The photons generated from the SPDC process in the BBO crystals therefore exhibit a wavelength of 780 nm. One photon of each created entangled photon pair (1,e and 2,e) is further sent to a PBS to create a three-photon entangled state (Hong-Ou-Mandel interference). Each of the three photons (1,e, 2,e and 1,o) enters one optical switch, respectively, that are driven by a square wave at 5 Hz with 50 % duty cycle. Therefore, as in the two-photon setup, half of the photons are sent to the verifier side and the other half to the user side. The measurement settings at the verifier side are randomly chosen by a quantum random number generator (QRNG). The photons are detected by APDs and their coincidences are recorded by a time tagger. The fourth photon (2,o) is triggered in horizontal (H) polarization via another PBS and serves as a trigger photon. Again, the user can perform any arbitrary measurement. Abbreviations: BBO, Beta Barium Borate; THWP, true zero-order half waveplate; YVO_4, Yttrium orthovanadate; $LiNbO_3$, Lithium niobate; OS, optical switch; HWP, half-wave plate; QWP, quarter-wave plate; PBS, polarizing beam splitter; APD, Avalanche Photodiodes; IF, interference filter;

where the indices e and o denote an (extra-) ordinary photon. The THWP further rotates this state into $|V\rangle_e|H\rangle_o$, while the pumping beam remains unchanged and is used to pump the second BBO, that also creates a photon pair in the state $|H\rangle_e|V\rangle_o$. After applying temporal and spatial compensations, the two possible ways of generating twin photons become indistinguishable. Hence, the entangled state $\frac{1}{\sqrt{2}}(|H\rangle_e|V\rangle_o - |V\rangle_e|H\rangle_o)$ is generated. In our case, we use two such sandwich-like entanglement sources. In order to generate a multipartite GHZ state, we transform the state described above into $\frac{1}{\sqrt{2}}(|HH\rangle + |VV\rangle)$ in the first source. We then take a single photon, prepared in the state $\frac{1}{\sqrt{2}}(|H\rangle + |V\rangle)$, from the second source. We do so by triggering one photon of the pair into horizontal polarization state and then applying local unitary to other heralded photon. By overlapping this heralded single photon with the entangled two-photon state at a polarizing beam splitter (PBS), the three-photon state $\frac{1}{\sqrt{2}}(|HHH\rangle + |VVV\rangle)$ is created via Hong-Ou-Mandel (HOM) interference. A filter with a bandwidth of 3 nm is inserted in each photon's path. After the photons enter the optical switches (OS) they are randomly sent to the verifier or the user side. Each side consists of an arrangement of quarter- and half-wave plates, PBSs and Avalanche photodiodes (APD) to analyze the polarization of the photons and to measure in any arbitrary basis. On the verifier's side, the waveplates are rotated to one of two measurements based on the output of a commercial quantum random number generator (QRNG) from ID quantique.

C. Synchronization of the optical switches

In order to synchronize the optical switches, we have to ensure that we modulate the OSs in phase. To achieve this, the outputs of the function generator producing the square signals driving the switches are synchronized. Synchronization can be achieved by observing both the wanted coincidence counts (those between detectors only in the verifier or the user side) and the unwanted cross coincidence counts (those occurring between detectors in the user and verifier's sides). By adjusting the phase between the square signals accordingly, the desired coincidence counts are maximized, while the unwanted cross events are minimized. The corresponding resulting coincidence events are shown in Fig. S3. Note that here all possible cross coincidences have been summed.

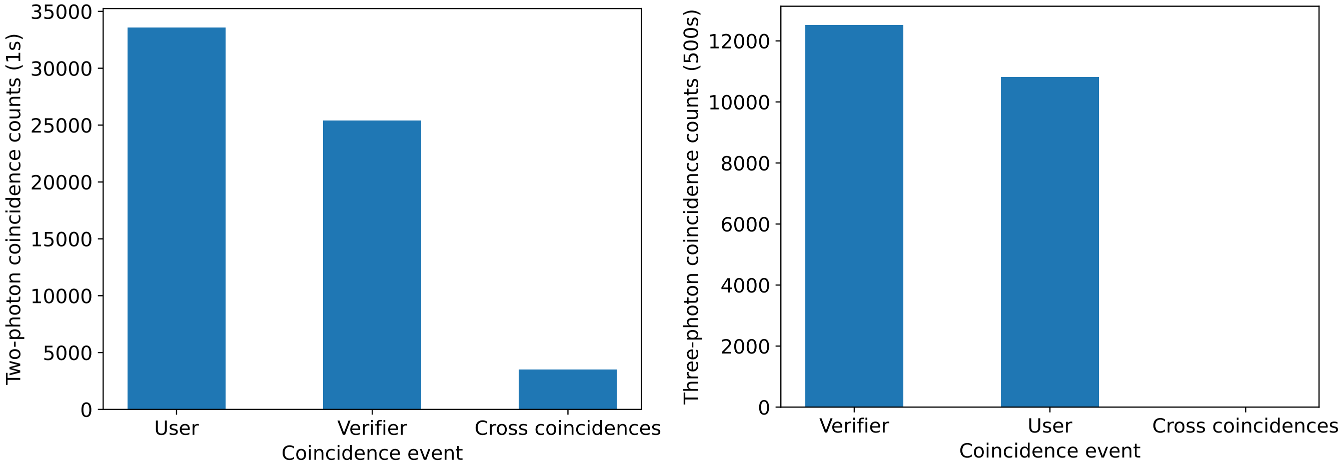

Figure S3: **Synchronization of the optical switches.** Coincidence counts between the different detectors for the two-photon setup (left) and three-photon setup (right). Coincidences were detected between channels on the user side, on the verifier side and cross coincidences are those between both sides.

D. Randomness quantification

The assumption that each state is randomly distributed between verifier and user is crucial for quantum state certification. In order to provide evidence that a given sequence of events is truly random, there exists a series of random number generation tests according to the National Institute of Standards and Technology (NIST) [55]. Using this tool, a sequence of binary bits is said to be randomly generated with a certain confidence if it passes all the tests.

In our case, the random distribution between the user and verifier results from the inherent randomness in the SPDC process, which we take advantage of by setting the OSs to switch faster than the multiphoton events occur. To generate a random bit sequence from our experiment, we assign a (multiphoton) coincidence event on the verifier side to '1' and a (multiphoton) coincidence event on the user side to '0'. However, the distribution of both the two-photon Bell state and the three-photon GHZ state between verifier and user is slightly biased, primarily due to different detection efficiencies of the SPDs and different insertion losses between the OSs. To be precise, for the two-photon Bell state, the bias is around 43 % for the verifier and 57 % for the user. For the three-photon GHZ state, the bias is around 53 % for the verifier and 47 % for the user. Unfortunately, the NIST tests do not tolerate any bias. Therefore, we have to apply a randomness extractor that removes the bias but keeps the intrinsic randomness of the data. For this purpose, we decided to use the von Neumann extractor [73]. The von Neumann extraction is applied by assigning '0' to two subsequent bits '01' and '1' to two subsequent bits '10'. Subsequent bits '00' and '11' are discarded. This procedure effectively removes the bias and still keeps the intrinsic randomness. The resulting data string after applying the von Neumann extractor contains the extracted random data to which we apply the NIST randomness tests. Before applying the tests, we assume the null hypothesis that the data are random, which we wish to confirm. The tests are statistical and each one yields a p-value to demonstrate the strength we confirm or reject the null hypothesis with. In this case, the higher the p-value the better the randomness. To analyze the data we first set a certain significance level α. If p-value $\geq \alpha$, the test is passed and the null hypothesis is confirmed. We set α into 0.01, meaning that we can confirm randomness with 99 % confidence. One drawback of the von Neumann extractor is that it diminishes our datasets by more than half. The amount of data we collected for the three-photon

GHZ state after having applied von Neumann extraction is not enough to perform the ninth test of the NIST test suite 'Universal Statistical Test'. For this reason, there is one test missing in Fig. 2 in the main text.

E. Success probability

To determine the overall probability of success, we calculate the ratio between the correct results and the number of samples used so far. For each sample, we perform a measurement that is randomly selected from the four possible settings. The outcome is either correct or incorrect, resulting in the curves shown in Fig. S4. Each time the success probability decreases, an incorrect result has been detected. However, after enough rounds the curve asymptotically approaches P_{exp}, which is $P_{\text{exp,Bell}} \approx 0.847$ for the two-photon Bell state (Fig. S4, top) and $P_{\text{exp,GHZ}} \approx 0.977$ for the three-photon GHZ state (Fig. S4, bottom). The insets in Fig. S4 show the extended measurement of the success probability.

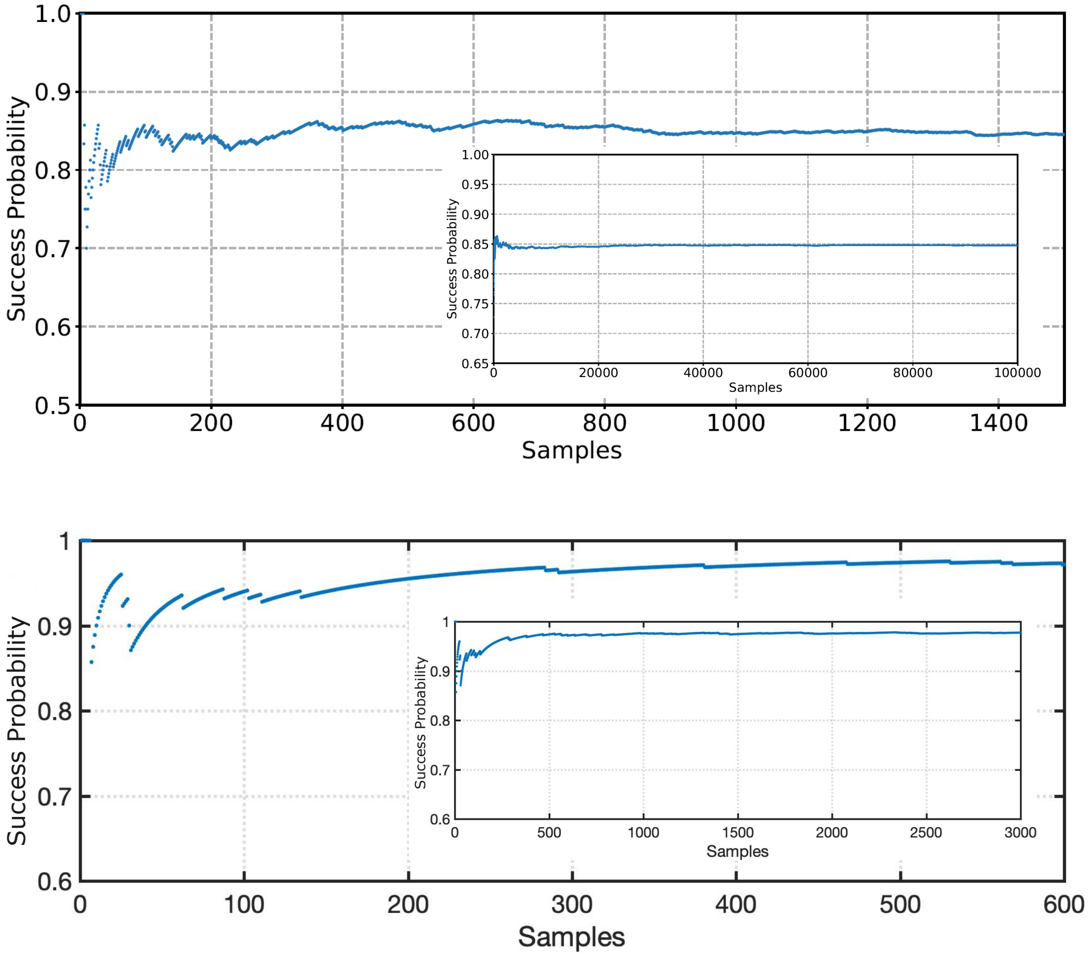

Figure S4: **Probability of success vs number of samples.** The upper panel shows the success probability in dependence of the number of samples for the two-photon Bell state, while the lower panel shows the same for the three-photon GHZ state. The insets in both figures show the extended measurements.

F. Fidelity estimation by Witness and self-testing

Device-dependent evaluation of fidelity by a witness.– We can determine the fidelity of the GHZ state by projecting it into the ideal GHZ state. The projective operator can be decomposed into local measurements, leading to a witness

in the form of [56, 74]

$$\mathcal{W}_{\rm GHZ} = |GHZ_3\rangle\langle GHZ_3| = \frac{1}{2}A_3 + \frac{1}{6}\sum_{k=1}^{3}(-1)^k M_k \tag{S.1}$$

$$A_3 = (|H\rangle\langle H|)^{\otimes 3} + (|V\rangle\langle V|)^{\otimes 3} \tag{S.2}$$

$$M_k = \left[\cos(\frac{k\pi}{3})\sigma_x + \sin(\frac{k\pi}{3})\sigma_y\right]^{\otimes 3}, \; k = 0,1,2 \tag{S.3}$$

The fidelity of a physical state ρ on the user side can be estimated by the evaluation of witness $F = \langle GHZ_3|\rho|GHZ_3\rangle = \langle W_{\rm GHZ}\rangle = 0.9679 \pm 0.0052$.

Device-independent valuation of fidelity by self-testing.–In a Bell inequality, entangled states can reach a maximal the value, called the quantum bound β_Q, which exceeds the classical bound $\beta_C < \beta_Q$ admitted by local hidden variable model. Experimentally, one typically observes a Bell value $\langle\mathcal{B}\rangle$ lower than the quantum bound, $\langle\mathcal{B}\rangle = \beta_Q - \epsilon$. In this case, robust self-testing can be used to provide a lower bound on the fidelity $\mathcal{F} = 1 - f(\epsilon)$ between the physical state and a target state up to local isometries. Endeavors have been made to optimize the function f to improve the robustness performance. Here we take use of the robust self-testing strategy of Ref. [41, 42] In the bipartite case, the underlying inequality for self-testing is Clauser-Horne-Shimony-Holt (CHSH) inequality

$$\mathcal{B}_{\rm Bell} = \langle A_0B_0\rangle + \langle A_0B_1\rangle + \langle A_1B_0\rangle - \langle A_1B_1\rangle < 2 \tag{S.4}$$

where $A_0 = X, A_1 = Z$ and $B_0 = \frac{X+Z}{\sqrt{2}}, B_1 = \frac{X-Z}{\sqrt{2}}$. The corresponding relation between the DI-fidelity lower bound and the observed Bell violation is given by

$$\mathcal{F}_{\rm Bell} \leq \frac{1}{2} + \frac{1}{2}\frac{\langle\mathcal{B}_{\rm Bell}\rangle - \beta^*}{2\sqrt{2} - \beta^*}, \quad \beta^* = \frac{16 + 14\sqrt{2}}{17} \approx 2.11 \tag{S.5}$$

In tripartite case, we apply the Mermin inequality

$$\mathcal{B}_{\rm GHZ} = \langle A_0B_0C_0\rangle + \langle A_0B_1C_1\rangle + \langle A_1B_0C_1\rangle - \langle A_1B_1C_0\rangle < 2 \tag{S.6}$$

where $A_0 = B_0 = C_0 = X$ and $A_1 = B_1 = -C_1 = -Y$. Then the relation between this observed violation and the DI-fidelity is

$$\mathcal{F}_{\rm GHZ} = \frac{1}{2} + \frac{1}{2}\frac{\langle\mathcal{B}_{\rm GHZ}\rangle - \gamma^*}{4 - \gamma^*}, \quad \gamma^* = 2\sqrt{2}. \tag{S.7}$$

G. Confidence Approximation

The confidence level $1-\delta$ provided by the original QSC protocol is $1 - \delta = 1 - \left[1 - \mu + \mu e^{-D}\right]^N$, where the $D = D(P_{\rm exp} \parallel P_\eta)$ and all other notations are defined in the main text. In our work, we instead use an approximation, $1 - \delta = 1 - e^{-D\mu N}$, to facilitate experimental implementation under realistic conditions. This approximation is convenient for practical use while maintaining sufficient accuracy.

We demonstrate that the two formulas are equivalent when higher-order terms of their Taylor expansions are omitted. The original definition can be approximated as

$$\begin{aligned}\delta &= \left[1 + \mu(e^{-D} - 1)\right]^N \\ &= 1 + N(e^{-D} - 1) + o(\mu^2[e^{-D} - 1]^2) \\ &= 1 + \mu N(-D) + o(D^2) + o(\mu[e^{-D} - 1]^2) \\ &\approx 1 - \mu ND\end{aligned} \tag{S.8}$$

Similarly, our approximation can be expressed as

$$\delta = e^{-D\mu N} = 1 - \mu ND + o([\mu ND]^2) \approx 1 - \mu ND \tag{S.9}$$

The advantage of the approximation is that the verifier can draw the conclusion merely based on his received copy numbers μN, without needing detailed knowledge of the ratio μ or the total sample number N.

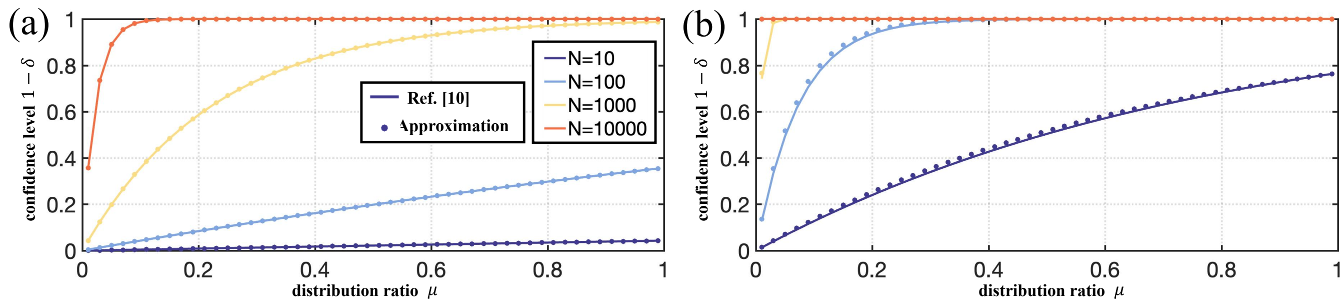

Figure S5: **Comparison of approximate confidence calculation with the original formula.** (a) We adopt the our experimental specification $P_{\text{exp,GHZ}} = 0.977$ and predetermined $P_\eta = 0.96$ aim to certify. We varied the distribution ratio μ from 0.01 to 0.99. The lines in our plots represent the results using our approximate formula, while the dots correspond to the results obtained from the original formula of Ref. [21]. We analyzed the results for $N = \{10, 100, 1000, 10000\}$. In each case, our approximate formula closely matches the original formula, validating the effectiveness of our approximation for both small and large sample sizes. (b) comparison under the parameters $P_{\text{exp,GHZ}} = 0.977$ and predetermined $P_\eta = 0.8$.

We also conducted numerical comparisons of the two definitions under various parameters, as shown in Fig. S5(a). By adopting our experimentally observed success probability $P_{\text{exp,GHZ}} = 0.977$ and the fidelity to be certified $P_\eta = 0.96$, we found that the results of the two formulas align well across a distribution ratio $\mu \in (0, 1)$, in both small ($N = 10$) and large ($N = 10000$) sample regimes. Notice that the approximation holds well when the experimentally observed winning probability P_{exp} is close to the p_η. This condition is naturally satisfied in most implementations, since the primary use-case of certification is for cases wherein the experimentally produced state should be close to a target state. i.e. we want to certify that a source is performing as designed. In this case, p_η will be close to P_{exp}. In any case, in Fig. S5 (b), we also show that even if a predetermined P_η is considerably far away from P_{exp}, the deviation of approximation from the exact expression remains small.

REFERENCES

1. N. Gisin, G. Ribordy, W. Tittel, H. Zbinden, Quantum cryptography. *Rev. Mod. Phys.* **74**, 145–195 (2002).

2. D. Deutsch, A. Ekert, Quantum computation. *Phys. World* **11**, 47–52 (1998).

3. D. F. V. James, P. G. Kwiat, W. J. Munro, A. G. White, Measurement of qubits. *Phys. Rev. A* **64**, 052312 (2001).

4. D. H. Mahler, L. A. Rozema, A. Darabi, C. Ferrie, R. Blume-Kohout, A. M. Steinberg, Adaptive quantum state tomography improves accuracy quadratically. *Phys. Rev. Lett.* **111**, 183601 (2013).

5. R. J. Chapman, C. Ferrie, A. Peruzzo, Experimental demonstration of self-guided quantum tomography. *Phys. Rev. Lett.* **117**, 040402 (2016).

6. B. Qi, Z. Hou, Y. Wang, D. Dong, H.-S. Zhong, L. Li, G.-Y. Xiang, H. M. Wiseman, C.-F. Li, G.-C. Guo, Adaptive quantum state tomography via linear regression estimation: Theory and two-qubit experiment. *NPJ Quant. Inf.* **3**, 19 (2017).

7. D. Gross, Y.-K. Liu, S. T. Flammia, S. Becker, J. Eisert, Quantum state tomography via compressed sensing. *Phys. Rev. Lett.* **105**, 150401 (2010).

8. S. T. Flammia, Y.-K. Liu, Direct fidelity estimation from few Pauli measurements. *Phys. Rev. Lett.* **106**, 230501 (2011).

9. C. Greganti, T. F. Demarie, M. Ringbauer, J. A. Jones, V. Saggio, I. A. Calafell, L. A. Rozema, A. Erhard, M. Meth, L. Postler, R. Stricker, P. Schindler, R. Blatt, T. Monz, P. Walther, J. F. Fitzsimons, Cross-verification of independent quantum devices. *Phys. Rev. X* **11**, 031049 (2021).

10. D. Zhu, Z. P. Cian, C. Noel, A. Risinger, D. Biswas, L. Egan, Y. Zhu, A. M. Green, C. H. Alderete, N. H. Nguyen, Q. Wang, A. Maksymov, Y. Nam, M. Cetina, N. M. Linke, M. Hafezi, C. Monroe, Cross-platform comparison of arbitrary quantum states. *Nat. Commun.* **13**, 6620 (2022).

11. M. Hayashi, Group theoretical study of LOCC-detection of maximally entangled states using hypothesis testing. *N. J. Phys.* **11**, 043028 (2009).

12. Y. Takeuchi, T. Morimae, Verification of many-qubit states. *Phys. Rev. X* **8**, 021060 (2018).

13. S. Pallister, N. Linden, A. Montanaro, Optimal verification of entangled states with local measurements. *Phys. Rev. Lett.* **120**, 170502 (2018).

14. H. Zhu, M. Hayashi, Optimal verification and fidelity estimation of maximally entangled states. *Phys. Rev. A* **99**, 052346 (2019a).

15. Z. Li, Y.-G. Han, H. Zhu, Optimal verification of Greenberger-Horne-Zeilinger states. *Phys. Rev. Appl.* **13**, 054002 (2020).

16. M. Hayashi,T. Morimae, Verifiable measurement-only blind quantum computing with stabilizer testing. *Phys. Rev. Lett.* **115**, 220502 (2015).

17. K. Fujii, M. Hayashi, Verifiable fault tolerance in measurement-based quantum computation. *Phys. Rev. A* **96**, 030301 (2017).

18. M. Hayashi, M. Hajdušek, Self-guaranteed measurement-based quantum computation. *Phys. Rev. A* **97**, 052308 (2018).

19. A. Dimić, B. Dakić, Single-copy entanglement detection. *NPJ Quant. Inf.* **4**, 11 (2018).

20. V. Saggio, A. Dimić, C. Greganti, L. A. Rozema, P. Walther,B. Dakić, Experimental few-copy multipartite entanglement detection. *Nat. Phys.* **15**, 935–940 (2019).

21. A. Gočanin, I. Šupić, B. Dakić, Sample-efficient device-independent quantum state verification and certification. *PRX Quant.* **3**, 010317 (2022).

22. W.-H. Zhang, C. Zhang, Z. Chen, X.-X. Peng, X.-Y. Xu, P. Yin, S. Yu, X.-J. Ye, Y.-J. Han, J.-S. Xu, G. Chen, C. F. Li, G. C. Guo, Experimental optimal verification of entangled states using local measurements. *Phys. Rev. Lett.* **125**, 030506 (2020).

23. J. Eisert, D. Hangleiter, N. Walk, I. Roth, D. Markham, R. Parekh, U. Chabaud, E. Kashefi, Quantum certification and benchmarking. *Nat. Rev. Phys.* **2**, 382–390 (2020).

24. Y.-G. Han, Z. Li, Y. Wang, H. Zhu, Optimal verification of the Bell state and Greenberger–Horne–Zeilinger states in untrusted quantum networks. *NPJ Quant. Inf.* **7**, 164 (2021).

25. H. Zhu, M. Hayashi, Efficient verification of pure quantum states in the adversarial scenario. *Phys. Rev. Lett.* **123**, 260504 (2019).

26. H. Zhu, M. Hayashi, General framework for verifying pure quantum states in the adversarial scenario. *Phys. Rev. A* **100**, 062335 (2019).

27. S. Barz, E. Kashefi, A. Broadbent, J. F. Fitzsimons, A. Zeilinger, P. Walther, Demonstration of blind quantum computing. *Science* **335**, 303–308 (2012).

28. W. Zhang, T. van Leent, K. Redeker, R. Garthoff, R. Schwonnek, F. Fertig, S. Eppelt, W. Rosenfeld, V. Scarani, C. C.-W. Lim, H. Weinfurter, A device-independent quantum key distribution system for distant users. *Nature* **607**, 687–691 (2022).

29. J. Yin, Y.-H. Li, S.-K. Liao, M. Yang, Y. Cao, L. Zhang, J.-G. Ren, W.-Q. Cai, W.-Y. Liu, S.-L. Li, R. Shu, Y. M. Huang, L. Deng, L. Li, Q. Zhang, N. L. Liu, Y. A. Chen, C. Y. Lu, X. B. Wang, F. Xu, J. Y. Wang, C. Z. Peng, A. K. Ekert, J. W. Pan, Entanglement-based secure quantum cryptography over 1,120 kilometres. *Nature* **582**, 501–505 (2020).

30. L.-M. Duan, M. D. Lukin, J. I. Cirac, P. Zoller, Long-distance quantum communication with atomic ensembles and linear optics. *Nature* **414**, 413–418 (2001).

31. D. P. Nadlinger, P. Drmota, B. C. Nichol, G. Araneda, D. Main, R. Srinivas, D. M. Lucas, C. J. Ballance, K. Ivanov, E. Y.-Z. Tan, P. Sekatski, R. L. Urbanke, R. Renner, N. Sangouard, J. D. Bancal, Experimental quantum key distribution certified by Bell's theorem. *Nature* **607**, 682–686 (2022).

32. M. Gimeno-Segovia, P. Shadbolt, D. E. Browne, T. Rudolph, From three-photon Greenberger-Horne-Zeilinger states to ballistic universal quantum computation. *Phys. Rev. Lett.* **115**, 020502 (2015).

33. P. Walther, K. J. Resch, T. Rudolph, E. Schenck, H. Weinfurter, V. Vedral, M. Aspelmeyer, A. Zeilinger, Experimental one-way quantum computing. *Nature* **434**, 169–176 (2005).

34. H. J. Briegel, D. E. Browne, W. Dür, R. Raussendorf, M. Van den Nest, Measurement-based quantum computation. *Nat. Phys.* **5**, 19–26 (2009).

35. R. Raussendorf, H. J. Briegel, A one-way quantum computer. *Phys. Rev. Lett.* **86**, 5188–5191 (2001).

36. J.-W. Pan, Z.-B. Chen, C.-Y. Lu, H. Weinfurter, A. Zeilinger, M. Z Żukowski, Multiphoton entanglement and interferometry. *Rev. Mod. Phys.* **84**, 777–838 (2012).

37. J.-W. Pan, D. Bouwmeester, M. Daniell, H. Weinfurter, A. Zeilinger, Experimental test of quantum nonlocality in three-photon Greenberger-Horne-Zeilinger entanglement. *Nature* **403**, 515–519 (2000).

38. G. L. Zanin, M. J. Jacquet, M. Spagnolo, P. Schiansky, I. A. Calafell, L. A. Rozema, P. Walther, Fiber-compatible photonic feed-forward with 99% fidelity. *Opt. Express* **29**, 3425–3437 (2021).

39. D. Mayers, A. Yao, Self testing quantum apparatus. arXiv:0307205 [quant-ph] (2004).

40. N. D. Mermin, Extreme quantum entanglement in a superposition of macroscopically distinct states. *Phys. Rev. Lett.* **65**, 1838–1840 (1990).

41. J. Kaniewski, Analytic and nearly optimal self-testing bounds for the Clauser-Horne-Shimony-Holt and Mermin inequalities. *Phys. Rev. Lett.* **117**, 070402 (2016).

42. W.-H. Zhang, G. Chen, X.-X. Peng, X.-J. Ye, P. Yin, Y. Xiao, Z.-B. Hou, Z.-D. Cheng, Y.-C. Wu, J.-S. Xu, C. F. Li, G. C. Guo, Experimentally robust self-testing for bipartite and tripartite entangled states. *Phys. Rev. Lett.* **121**, 240402 (2018).

43. I. Šupić, J. Bowles, Self-testing of quantum systems: A review. *Quantum* **4**, 337 (2020).

44. H. Chernoff, A measure of asymptotic efficiency for tests of a hypothesis based on the sum of observations. *Ann. Math. Statist.* **23**, 493–507 (1952).

45. W. Hoeffding, *The Collected Works of Wassily Hoeffding* (Springer Science & Business Media, 2012).

46. S. P. Neumann, M. Selimovic, M. Bohmann, R. Ursin, Experimental entanglement generation for quantum key distribution beyond 1 Gbit/s. *Quantum* **6**, 822 (2022).

47. C. Zhang, Y.-F. Huang, Z. Wang, B.-H. Liu, C.-F. Li, G.-C. Guo, Experimental Greenberger-Horne-Zeilinger-type six-photon quantum nonlocality. *Phys. Rev. Lett.* **115**, 260402 (2015).

48. S. Aerts, P. Kwiat, J.-A. Larsson, M. Zukowski, Two-photon franson-type experiments and local realism. *Phys. Rev. Lett.* **83**, 2872–2875 (1999).

49. J.-Å. Larsson, Loopholes in Bell inequality tests of local realism. *J. Phys. A. Math. Theor.* **47**, 424003 (2014).

50. L. Huang, X.-M. Gu, Y.-F. Jiang, D. Wu, B. Bai, M.-C. Chen, Q.-C. Sun, J. Zhang, S. Yu, Q. Zhang, C. Y. Lu, J. W. Pan, Experimental demonstration of genuine tripartite nonlocality under strict locality conditions. *Phys. Rev. Lett.* **129**, 060401 (2022).

51. D. R. Hamel, L. K. Shalm, H. Hübel, A. J. Miller, F. Marsili, V. B. Verma, R. P. Mirin, S. W. Nam, K. J. Resch, T. Jennewein, Direct generation of three-photon polarization entanglement. *Nat. Photonics* **8**, 801–807 (2014).

52. P. Walther, M. Aspelmeyer, A. Zeilinger, Heralded generation of multiphoton entanglement. *Phys. Rev. A* **75**, 012313 (2007).

53. H. Cao, L. M. Hansen, F. Giorgino, L. Carosini, P. Zahálka, F. Zilk, J. C. Loredo, P. Walther, Photonic source of heralded Greenberger-Horne-Zeilinger states. *Phys. Rev. Lett.* **132**, 130604 (2024).

54. L. K. Shalm, Y. Zhang, J. C. Bienfang, C. Schlager, M. J. Stevens, M. D. Mazurek, C. Abellán, W. Amaya, M. W. Mitchell, M. A. Alhejji, H. Fu, J. Ornstein, R. P. Mirin, S. W. Nam, E. Knill, Device-independent randomness expansion with entangled photons. *Nat. Phys.* **17**, 452–456 (2021).

55. L. E. Bassham, A. L. Rukhin, J. Soto, J. R. Nechvatal, M. E. Smid, S. D. Leigh, M. Levenson, M. Vangel, N. A. Heckert, D. L. Banks, A statistical test suite for random and pseudorandom number generators for cryptographic applications (National Institute of Standards and Technology, 2010).

56. Y.-F. Huang, B.-H. Liu, L. Peng, Y.-H. Li, L. Li, C.-F. Li, G.-C. Guo, Experimental generation of an eight-photon Greenberger–Horne–Zeilinger state. *Nat. Commun.* **2**, 546 (2011).

57. X.-C. Yao, T.-X. Wang, P. Xu, H. Lu, G.-S. Pan, X.-H. Bao, C.-Z. Peng, C.-Y. Lu, Y.-A. Chen, J.-W. Pan, Observation of eight-photon entanglement. *Nat. Photonics* **6**, 225–228 (2012).

58. J.-D. Bancal, *On the Device-Independent Approach to Quantum Physics: Advances in Quantum Nonlocality and Multipartite Entanglement Detection*, Springer Theses (Springer International Publishing, 2014), pp. 73–80.

59. M. K. Joshi, C. Kokail, R. van Bijnen, F. Kranzl, T. V. Zache, R. Blatt, C. F. Roos, P. Zoller, Exploring large-scale entanglement in quantum simulation. *Nature* **624**, 539–544 (2023).

60. J. Zhang, G. Pagano, P. W. Hess, A. Kyprianidis, P. Becker, H. Kaplan, A. V. Gorshkov, Z.-X. Gong, C. Monroe, Observation of a many-body dynamical phase transition with a 53-qubit quantum simulator. *Nature* **551**, 601–604 (2017).

61. A. L. Shaw, Z. Chen, J. Choi, D. K. Mark, P. Scholl, R. Finkelstein, A. Elben, S. Choi, M. Endres, Benchmarking highly entangled states on a 60-atom analogue quantum simulator. *Nature* **628**, 71–77 (2024).

62. T. M. Graham, Y. Song, J. Scott, C. Poole, L. Phuttitarn, K. Jooya, P. Eichler, X. Jiang, A. Marra, B. Grinkemeyer, M. Kwon, M. Ebert, J. Cherek, M. T. Lichtman, M. Gillette, J. Gilbert, D. Bowman, T. Ballance, C. Campbell, E. D. Dahl, O. Crawford, N. S. Blunt, B. Rogers, T.

Noel, M. Saffman, Multi-qubit entanglement and algorithms on a neutral-atom quantum computer. *Nature* **604**, 457–462 (2022).

63. S. Cao, B. Wu, F. Chen, M. Gong, Y. Wu, Y. Ye, C. Zha, H. Qian, C. Ying, S. Guo, Q. Zhu, H. L. Huang, Y. Zhao, S. Li, S. Wang, J. Yu, D. Fan, D. Wu, H. Su, H. Deng, H. Rong, Y. Li, K. Zhang, T. H. Chung, F. Liang, J. Lin, Y. Xu, L. Sun, C. Guo, N. Li, Y. H. Huo, C. Z. Peng, C. Y. Lu, X. Yuan, X. Zhu, J. W. Pan, Generation of genuine entanglement up to 51 superconducting qubits. *Nature* **619**, 738–742 (2023).

64. L. D. S. Martins, N. Laurent-Puig, I. Šupić, D. Markham, E. Diamanti, Experimental sample-efficient and device-independent GHZ state certification. arXiv:2407.13529 [quant-ph] (2024).

65. B. Hensen, H. Bernien, A. E. Dréau, A. Reiserer, N. Kalb, M. S. Blok, J. Ruitenberg, R. F. L. Vermeulen, R. N. Schouten, C. Abellán, W. Amaya, V. Pruneri, M. W. Mitchell, M. Markham, D. J. Twitchen, D. Elkouss, S. Wehner, T. H. Taminiau, R. Hanson, Loophole-free Bell inequality violation using electron spins separated by 1.3 kilometres. *Nature* **526**, 682–686 (2015).

66. L. K. Shalm, E. Meyer-Scott, B. G. Christensen, P. Bierhorst, M. A. Wayne, M. J. Stevens, T. Gerrits, S. Glancy, D. R. Hamel, M. S. Allman, K. J. Coakley, S. D. Dyer, C. Hodge, A. E. Lita, V. B. Verma, C. Lambrocco, E. Tortorici, A. L. Migdall, Y. Zhang, D. R. Kumor, W. H. Farr, F. Marsili, M. D. Shaw, J. A. Stern, C. Abellán, W. Amaya, V. Pruneri, T. Jennewein, M. W. Mitchell, P. G. Kwiat, J. C. Bienfang, R. P. Mirin, E. Knill, S. W. Nam, Strong loophole-free test of local realism. *Phys. Rev. Lett.* **115**, 250402 (2015).

67. M. Giustina, M. A. M. Versteegh, S. Wengerowsky, J. Handsteiner, A. Hochrainer, K. Phelan, F. Steinlechner, J. Kofler, J.-Å. Larsson, C. Abellán, W. Amaya, V. Pruneri, M. W. Mitchell, J. Beyer, T. Gerrits, A. E. Lita, L. K. Shalm, S. W. Nam, T. Scheidl, R. Ursin, B. Wittmann, A. Zeilinger, Significant-loophole-free test of Bell's theorem with entangled photons. *Phys. Rev. Lett.* **115**, 250401 (2015).

68. A. Cabello, D. Rodríguez, I. Villanueva, Necessary and sufficient detection efficiency for the mermin inequalities. *Phys. Rev. Lett.* **101**, 120402 (2008).

69. X.-s. Ma, S. Zotter, N. Tetik, A. Qarry, T. Jennewein, A. Zeilinger, A high-speed tunable beam splitter for feed-forward photonic quantum information processing. *Opt. Express* **19**, 22723 (2011).

70. P. Wang, S. Baek, M. Yabuno, S. Miki, H. Terai, F. Kaneda, Low-loss polarization-maintaining router for single and entangled photons at a telecom wavelength. arXiv:2502.12554 [quant-ph] (2025).

71. W.-Z. Liu, M.-H. Li, S. Ragy, S.-R. Zhao, B. Bai, Y. Liu, P. J. Brown, J. Zhang, R. Colbeck, J. Fan, Q. Zhang, J. W. Pan, Device-independent randomness expansion against quantum side information. *Nat. Phys.* **17**, 448–451 (2021).

72. W.-Z. Liu, Y.-Z. Zhang, Y.-Z. Zhen, M.-H. Li, Y. Liu, J. Fan, F. Xu, Q. Zhang, J.-W. Pan, Toward a photonic demonstration of device-independent quantum key distribution. *Phys. Rev. Lett.* **129**, 050502 (2022).

73. Y. Peres, Iterating von Neumann's procedure for extracting random bits. *Ann. Stat.* **20**, 590 (1992).

74. O. Gühne, C.-Y. Lu, W.-B. Gao, J.-W. Pan, Toolbox for entanglement detection and fidelity estimation. *Phys. Rev. A* **76**, 030305 (2007).
